# The marginal causal effect of opium consumption on the upper gastrointestinal cancer death using parametric g-formula: An analysis of 49,946 cases in the Golestan Cohort Study, Iran

**DOI:** 10.1371/journal.pone.0246004

**Published:** 2021-01-25

**Authors:** Neda Mohammadi, Masoomeh Alimohammadian, Akbar Feizesani, Hossein Poustchi, Ahad Alizadeh, Mehdi Yaseri, Mohammad Ali Mansournia, Alireza Sadjadi

**Affiliations:** 1 Department of Epidemiology and Biostatistics, School of Public Health, Tehran University of Medical Sciences, Tehran, Iran; 2 Digestive Oncology Research Center, Digestive Disease Research Institute, Tehran University of Medical Sciences, Tehran, Iran; 3 Department of Human Ecology, School of Public Health, Tehran University of Medical Sciences, Tehran, Iran; 4 Metabolic Diseases Research Center, Research Institute for Prevention of Non-Communicable Diseases, Qazvin University of Medical Sciences, Qazvin, Iran; 5 No Way New Way Company, The Hague, The Netherlands; National Yang-Ming University School of Medicine, TAIWAN

## Abstract

Upper gastrointestinal (UGI) cancer, including esophageal and gastric, is one of the most common cancers in the world. Hence, the determination of risk factors of UGI helps to reduce the economic and social burden of this cancer in communities. In Iran, the consumption of opium because of its neighborhood with Afghanistan are considerable. In this study, we examine the causal effect of opium use on the time to UGI cancer death. Based on the Golestan Cohort Study (GCS) in northeastern of Iran, about 50000 adults were enrolled to the study for four years (2004–2008) and followed annually until July 2018. We used “parametric g-formula” to study the causal effect of opium use on the time to death due to UGI. In this study, the information of 49946 individuals due to missingness were analyzed. So the median of follow-up time was 144 months and the prevalence of opium use was 17% (about 8489 persons). During the follow-up period, 593 (1.2%) death from upper gastrointestinal cancer were reported. The study showed that the effect of opium use on the time to UGI death was statistically significant (adjusted risk-ratio based on parametric g-formula = 1.31, 95% CI: [1.04, 1.65]). Additionally, the Population Attributable Fraction (PAF) in UGI cancer deaths of opium use was estimated 5.3% (95% CI: [0.6%, 11.3%]). Our results showed a causal effect of opium use on the intensity of upper gastrointestinal cancer death.

## Introduction

According to the World Health Organization (WHO), in 2016, 71% of all deaths were related to Non-communicable diseases that 22% of such deaths were caused by cancers [1]. Upper gastrointestinal cancers (including stomach cancer and esophageal cancer) are among the prevalent cancers. Stomach cancer is the fifth and esophageal cancer is the eighth leading cause of death in the world. In Iran, according to the estimated report from GLOBOCAN 2018, stomach and esophageal cancer are the first and ninth leading cause of death, respectively [2] and the age-standardized mortality rate (per 100,000 population) for stomach and esophageal cancer are 12.3 and 2.9, respectively [2]. It has been estimated that 50% of cancers are preventable by modifying cancer risk factors from people’s lifestyles, so knowing about the risk factors of cancers is essential [3]. Regular uses of opium have been discerned as a risk factor for many disease and cancers, but there are a few studies have pointed to the association between opium use and upper gastrointestinal cancer [4, 5]. Opium is the air-dried extract of the opium poppy, Papaver somniferum. In 2008, about 13–22 million people, mostly from Asian countries, used opium and its derivatives [6]. Smoking opium was limited to China and some other countries in South-Eastern of Asia [7]. But currently, opium is consumed by traditional means in many third world countries where it is grown. In Iran, The most common opioids ever used was opium [8].

There are some researches about the effect of opium use on types of cancer that all of them used the Cox proportional hazard model and estimated hazard ratio (HR) as their statistical tool to obtain the relation while the Cox model has two limitations.

The first limitation is that even in the absence of confounders and measurement errors, there are three reasons that the hazard ratio in causal inference is not an appropriate index. The first reason is that when outcomes are the time to an event, the hazard ratio is not collapsible. It means that there is a discrepancy between the marginal HR and the conditional HR even in the absence of confounding [9–11]. Second, the time-specific HR has a built-in selection bias because in each time we condition on individuals who survived in the previous time, so we made an unreal relationship between the exposure and risk factors of outcome only on survived subjects [9, 12, 13]. Finally, hazards might vary over time, so one could expect that the hazard ratio should change over time. However, due to the first limitation of the Cox proportional hazards model, many published studies in survival settings report a single hazard ratio and ignore the interactions with time [14].

Another limitation is that the Cox model is a conditional model. Usually, the study's target is to find the effect of an exposure (like opium) on the outcome in the total population. In these situations, the effect at the individual level is not of interest. Marginal effects models would be preferable because they find the effect in the total population [15].

Because of the mentioned reasons, the Cox regression model and Hazard Ratio index are not appropriate in a causal setting.

This study aimed to estimate the marginal causal effect of opium on upper gastrointestinal cancer death using a more appropriate model named parametric g-formula that reports suitable marginal index, Relative Risk (RR), as an effect size. The marginal models can estimate the causal effect of exposure if all people in the population use exposure against when all people do not use it using pseudo population.

## Materials and methods

### Study population and measurements

Details on the design of this study have been reported in the Golestan cohort profile [16]. In summary, the Golestan Cohort Study (GCS) is a prospective cohort study. It was launched in January 2004 and study participants were selected using a random sampling of persons without a history of upper gastrointestinal cancer who lived in the Golestan province, northeast of Iran and in June 2008 the goal of 50,000 subjects was reached and the enrollment was closed.

Each participant was interviewed by a trained general physician and a trained nutritionist to collect data on lifestyle and medical history using a general questionnaire at baseline.

The outcome was UGI cancer death. To get information about outcome, all participants are being followed up by telephone calls annually. If a death is reported by family members, friends, or local health workers, the physician uses a validated verbal autopsy questionnaire to interview the closest relative of the deceased.

The exposure was the usage of opium. In this study, opium use was defined as opium consumption at least once a week for six months. In this regard, the subjects were divided into two groups of non-opium users and opium users. The opiate types that were included in this study were Teriak (raw opium), Sukhteh, Shireh, and heroin. Sukhteh is the dry, black residue of smoked Teriak, which is scraped from opium pipe. Shireh is a refined opium product made by boiling a combination of opium and Sukhteh in hot water and passing the solution through filters several times [6].

Other risk factors which considered in this study were gender (male/female), age of interview (years), body mass index (BMI) at baseline (Underweight as BMI<18.5 /Normal between 18.5 to 25/ Overweight 25 to 30/Obese as BMI≥30), ethnicity (Turkmen, non-Turkmen), education (Illiterate/up to 8 years/high school/university), residential place (urban/rural), vegetables and fruits intake per day (lower than 400g/upper than 400g), smoking(yes/no), tobacco smoking(yes/no), alcohol consumption (yes/no), marital status(married/otherwise). Socioeconomic status was classified into three categories (low/middle/high). To build a composite score for socioeconomic status, principal components analysis (PCA) was used on the ownership of refrigerator, freezer, computer, dishwasher, color TV, LCD, motorcycle, car, personal bathroom and vacuum cleaner and then this score was categorized into three equal percentile from “poor” to “rich”.

### Statistical methods

The goal of most studies, including medical studies is to investigate the causal effect of the treatment/exposure variable on the outcome. For estimating causal effects, randomized experiments are generally considered the gold standard. But when randomization is not feasible because of ethical concerns, excessive expense, or timeliness, researchers use non-randomized observational studies [17]. Due to non-randomly allocation, Observational studies are prone to confounding bias and in these studies, association does not ensure causation [18, 19]. Many causal problems are concerned with treatment effects on time to occurrence an event like the death in the survival setting.

In this study, we use the “parametric g-formula” to estimate the causal effect and report the risk-ratio index of opium on the time to death due to UGI. Parametric g-formula is a class of g-methods that can be used for any generalized time fixed or time-varying treatment [14, 20, 21].

Let *A* denote an exposure (A = 1 for opium users and A = 0 for non-opium users), *k* indicates the time of follow-up, L vector of confounders (other risk factors) and *D*_*k*_ denotes the binary variable which indicates the event at time K (happened = 1, not happened = 0). The steps to implement the parametric g-formula have been described in detail elsewhere [14]. G-formula fitting procedure as follows: first, we extend data in the person-time data format. It means that each row of the dataset corresponds to a person-time and the first row contains details about the first person at time = o, the second row contains information about the same person in time = 1 and so on till the end of follow up for person 1. Then the information of other subjects would be completed in the same manner after person 1. In the second step, we calculate conditional survival *P*(*D*_*k*+1_ = 0|*L* = *l,A* = *a*). To obtain it, we fit a hazard model for example by$logit\;P(D_{k+1}=1|D_{k}=0,A=a,L=l)=\theta_{0,k}+\theta_{1}A+\theta_{2}A\times k+\theta_{3}L$.

Then, we allocate all participants to treatment and also the non-treatment group as a counterfactual concept. To do this, we copy all confounders' distribution for both of the treatment situations and calculate the probability. And then using $P(D_{k+1}=0|L=l,A=a)=\prod_{m=1}^{k}P(D_{m+1}=0|D_{m}=0,L=l,A=a)$ we calculated conditional hazard. In the third step, to reach the marginal causal RR, we standardize survival on confounder distribution using $\sum_{l}P(D_{k+1}=0|L=l,A=a)\times P(L=l)$ for treatment and non-treatment groups, so we calculated risk using 1-survival in each time and eventually, we got the $causal\;RR=\frac{\sum_{l}P(D_{k+1}=1|L=l,A=1)\times P(L=l)}{\sum_{l}P(D_{k+1}=1|L=l,A=0)\times P(L=l)}$.

Another measure that we studied was Population Attributable Fraction (PAF) to investigate the impact of opium. It is the estimated fraction of all cases that would not have occurred if there had been no exposure. Using a cohort study, following Miettinen, we estimated the PAF from the estimated relative risk (RR) for the exposure and the prevalence of exposure among cases (pc), as PAF = pc(1 −1/RR) [22].

The statistical analysis was performed using SAS software (version 9.4). 95% confidence intervals were constructed based on 1000 bootstrap replications.

## Results

During a median follow-up of 144 months (3–180 months), 8489 participants (about 17%) were opium users. The frequency of consumption in derivatives of opium between participants was: Teriak 8345 (86%), Shireh 1243 (12.8%), Sukhteh 11 (0.1%), and Heroin 109 (1.1%). Demographic information for opium users and non-users has shown in **Table 1**.

**Table 1:** Baseline characteristics of the Golestan Cohort participants, Iran.

		Total	Opium user	
		(N = 49946)	No (N = 41457)	Yes (N = 8489)
Age of interview [Mean(SD)]		52.12 (9.02)	51.8 (8.93)	53.68 (9.26)
Gender	Female	28748 (57.6%)	26397 (63.7%)	2351 (27.7%)
	Male	21198 (42.4%)	15060 (36.3%)	6138 (72.3%)
Ethnicity	Torkmen	37253 (74.6%)	30693 (74%)	6560 (77.3%)
	Other	12693 (25.4%)	10764 (26%)	1929 (22.7%)
SES	Rich	16566 (33.2%)	14603 (35.2%)	1963 (23.1%)
	Middle	17051 (34.1%)	14240 (34.4%)	2811 (33.1%)
	Poor	16327 (32.7%)	12612 (30.4%)	3715 (43.8%)
Residential areas	Urban	11592 (23.2%)	10244 (24.7%)	1348 (15.9%)
	Rural	38354 (76.8%)	31213 (75.3%)	7141 (84.1%)
BMI	Underweight	2380 (4.8%)	1314 (3.2%)	1066 (12.6%)
	Normal	17871 (35.8%)	13515 (32.6%)	4356 (51.3%)
	Overweight	16993 (34%)	14900 (35.9%)	2093 (24.7%)
	Obese	12694 (25.4%)	11723 (28.3%)	971 (11.4%)
Education	Illiterate	35060 (70.2%)	29434 (71%)	5626 (66.3%)
	Up to 8 year	10684 (21.4%)	8452 (20.4%)	2232 (26.3%)
	High school	3141 (6.3%)	2588 (6.2%)	553 (6.5%)
	University	1061 (2.1%)	983 (2.4%)	78 (0.9%)
Vegetables intake/day	Upper than 400mg	9263 (18.9%)	7794 (19.1%)	1469 (17.6%)
	Lower than 400mg	39785 (81.1%)	32922 (80.9%)	6863 (82.4%)
Smoking	Never	41323 (82.7%)	37301 (90%)	4022 (47.4%)
	Ever	8622 (17.3%)	4156 (10%)	4466 (52.6%)
Tobacco consumption	No	45519 (91.1%)	39714 (95.8%)	5805 (68.4%)
	Yes	4427 (8.9%)	1743 (4.2%)	2684 (31.6%)
Alcohol use	No	48225 (96.6%)	40661 (98.1%)	7564 (89.1%)
	Yes	1721 (3.4%)	796 (1.9%)	925 (10.9%)
Marital status	Married	43873 (87.8%)	36318 (87.6%)	7555 (89%)
	Single	6073 (12.2%)	5139 (12.4%)	934 (11%)

All measurements are reported as N (%) unless otherwise was indicate.

In this study, all confounders were chosen based on previous reports and according to the definition of confounders, the effects of confounders on exposure and outcome were checked.

Weak confounders were removed using the stepwise method based on a 10% cutoff point criteria. According to the stepwise method, at each step, the covariate that causes the smallest change in the exposure effect on outcome is removed. The process stops when the deletion of each of the remaining variables causes a relative change of more than 10% cutoff value [23]. So we choose gender (male-female), age of interview (continuous variable), ethnicity (Turkmen, non-Turkmen), socioeconomic (low, middle, high), residential place (urban, rural), BMI (Underweight, Normal, Overweight, obese), education(Illiterate, up to 8 years, high school, university), vegetables and fruits intake per day (lower than 400g/upper than 400g), smoking (yes/no), tobacco consumption (yes/no), alcohol use (yes/no) and marital status (married/otherwise) as confounders.

During the follow-up, of the 7046 participants who died, 592 persons died from UGI cancer. We analyzed these data for 2 data sets: the first dataset includes all participants and the second one contains participants without anyone who died due to any reason other than UGI cancer. Using the g-formula method, we calculate the causal effect of opium on the time to UGI cancer death that is shown in **Table 2**.

**Table 2:** Adjusted risk ratio for causation between opium use and the UGI cancer death in the Golestan Cohort Study.

Dataset	opium use	No. of participants (No. of UGI-Deaths)	adjusted Risk Ratio^c^	95% CI
First^a^	Never users	41457 (417)	Reference	-
	Ever users	8489 (175)	1.31	1.04, 1.65
Second^b^	Never users	36954 (417)	Reference	-
	Ever users	6538 (175)	1.38	1.10, 1.65

^a^ Based on all participants information.

^b^ All participants were included except who died due to any reason other than UGI cancer.

^c^ Adjusted for gender, ethnicity, SES, area, BMI, age, time of follow up, education, vegetables intake, smoking, tobacco and alcohol consumption, marital status.

Parametric g-formula as a marginal model can estimate risk ratio if all people in the population use opium versus if all do not use it. It shows that: in the first dataset, if all people are opium user, the risk of UGI cancer death is 1.28 times to other situation that all people were opium non-users (adjusted RR = 1.31; 95%CI: [1.04, 1.65]) and also in the second dataset, if all people are opium user the risk of UGI cancer death is 1.38 times to other situation that all people were opium non-users (adjusted RR = 1.38; 95%CI: [1.10,1.65]).

**Fig 1:**
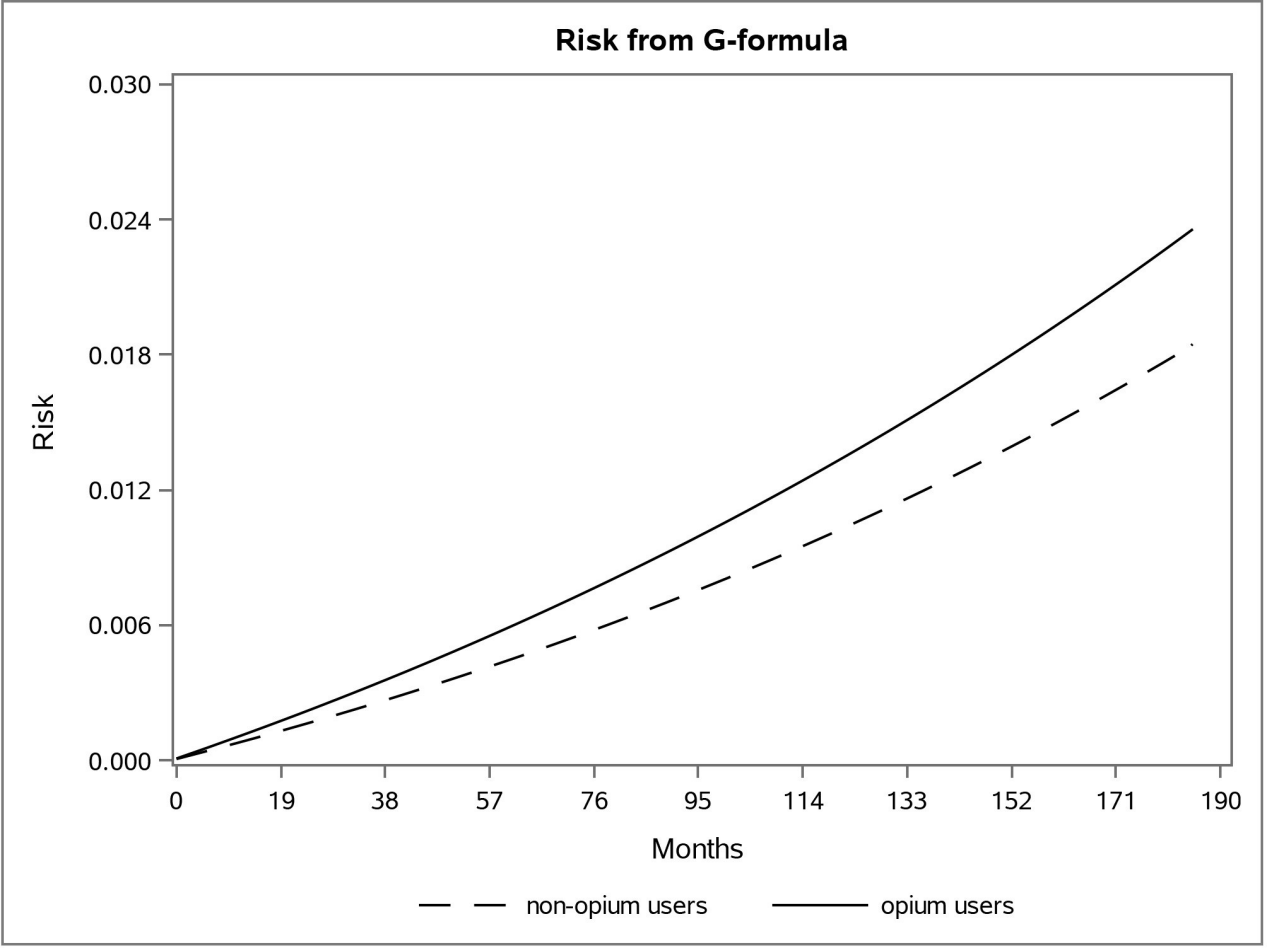
The adjusted risk graph using the parametric g-formula for two groups of opium users and non-opium users in the first dataset in the Golestan Cohort Study.

**Fig 2:**
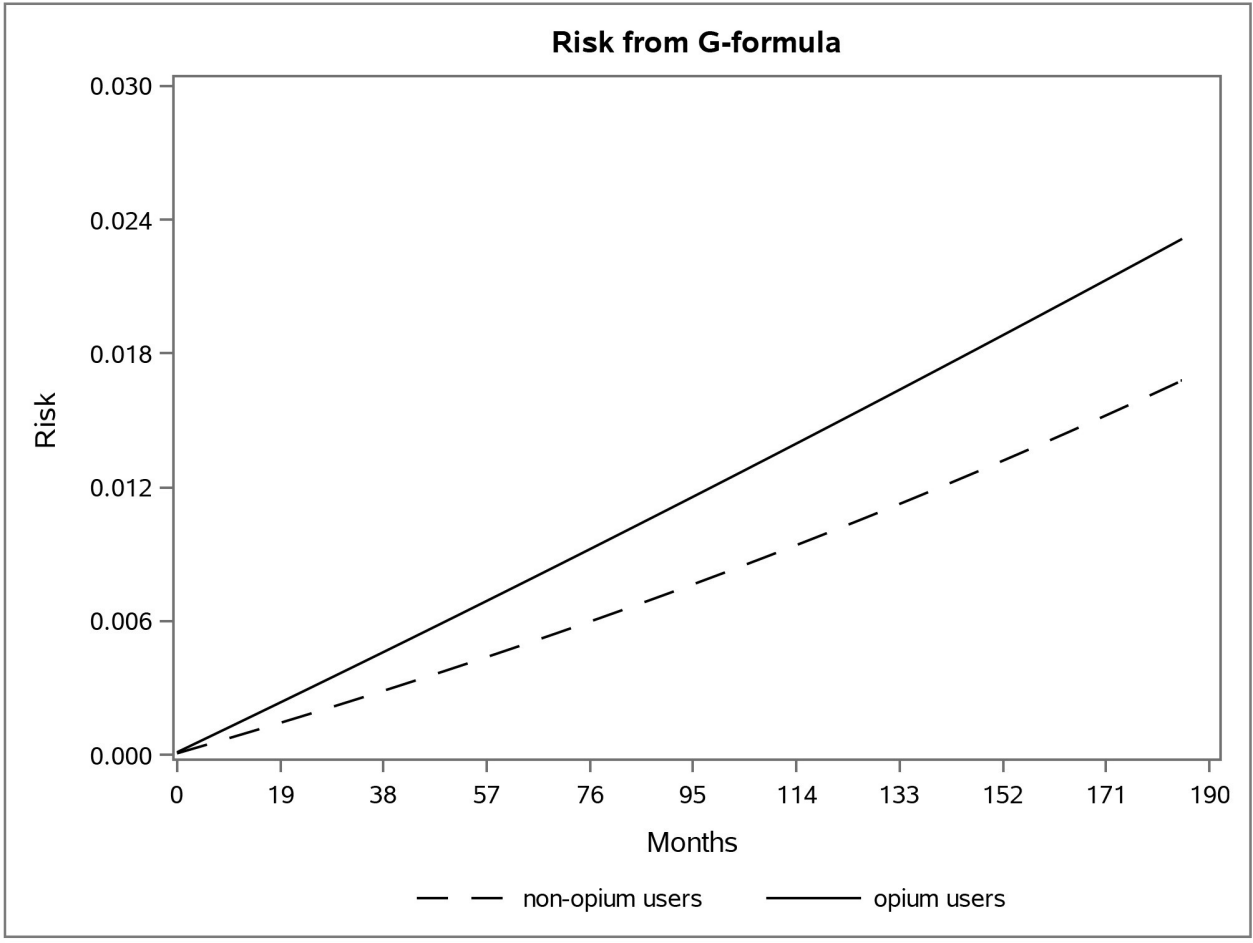
The adjusted risk graph using the parametric g-formula for two groups of opium users and non-opium users in the second dataset in the Golestan Cohort Study.

The adjusted survival curve using the g-formula method in the first and second data is shown in **Figs 1 and 2**.

We also calculated the population attributable fraction. The estimated fraction of UGI cancer death in the first dataset was about 5.33% (95% CI: [6.23, 11. 31]) and in the second one was 6.9% (95% CI: [0.71, 11.73]).

## Discussion

We aimed to examine the marginal causal effect of opium use on UGI cancer mortality using the g-formula method. In this study, 8489 (17%) of all participants were opium users. We found after adjusting for confounders, opium users had 1.28 times the risk of UGI cancer death compared to non-users (RR = 1.28; 95%CI: [1.04, 1.65]) and after elimination of anyone who died due to any reason other than UGI cancer, Opium users had 1.38 times the risk of UGI cancer death compared to non-users) RR = 1.38; 95%CI: [1.10, 1.65]). So this analysis showed the causal effect of opium use on time to death due to upper gastrointestinal cancer.

To the best of our knowledge, this paper was the first to study the causal effect of opium on UGI cancer using the marginal causal model. Our finding of a positive effect of opium consumption on UGI cancer death supports findings in previous studies, but all of the previous reports used conditional methods like logistic regression and Cox proportional hazard model. They have shown a positive association between opium use and cancers of the esophagus [4, 5, 24–26] and stomach [4–6, 27, 28], all of them report the significant association between opium use and gastric cancers.

Conditional methods like the Cox model estimate the conditional effect at the individual level and when the target of the intervention on the exposure (like opium) is the total population and the effect at the individual level is not of interest, we should use the marginal effects. Another problem in the Cox model is about the hazard-ratio. There are three reasons that the hazard ratio in causal inference is not an appropriate index. The first reason is that when outcomes are the time to an event, the hazard ratio is not collapsible means that there is a discrepancy between the marginal HR and the conditional HR even in the absence of confounding [9–11]. Also, the time-specific HR has a built-in selection bias. The hazard at time 2 is the probability of dying at time two among individuals who survived past time one, so because of this conditioning we made an unreal relationship between the exposure and factors that are just risk factors of outcome is referred to as frailty [12, 13]. The last reason is that hazards may vary over time, so the hazard ratio does too. But many published reports in survival settings report a single hazard ratio and ignore the interactions with time because of fitting the Cox proportional hazards model [14].

Because of these reasons, we used g-formula to estimate the marginal causal effect. G-formula is not the only method to estimate the marginal causal effect but also inverse probability weighting (IPW) does the same [29, 30] but it is a weight-base method that can be unstable in the presence of large weights.

In causal inference, exposure should be well-defined. In this study, we did not consider dosage and time of usage, but in fact, we examined the consumption of opium in the distribution of the Golestan community, so it is well-defined in this regard. Another assumption in causal inference is there should be no unmeasured confounding. In this study, we had chosen potential confounders based on the literature review. Then, according to the definition of the confounder, the selected potential confounders were evaluated based on the data. In addition, the selected confounders were excluded from the analysis if the presence of them could not vary the relative change of exposure’s effect on the outcome more than 10%. Except for the socioeconomic status variable, all variables were obtained by asking each participant according to the questionnaire. The SES variable was made on asset variables like ownership of refrigerator, freezer, computer, dishwasher, color TV, LCD, motorcycle, car, personal bathroom and vacuum cleaner and using principal components analysis (PCA), SES were grouped into three sections from “poor” to “rich”. However, despite all efforts, maybe there were some confounders that did not include in our model.

The advantages of this article were the large sample size, lack of loss to follow up, the prospective study, the availability of confounders information, and the latest update of Golestan Cohort data (July 2018). Also, the statistical method that we used was an excellent method to calculate the causal effect.

A possible extension of this work is to perform another analysis that includes the dosage and time of usage of opium.

In this study, based on the parametric g-formula method, we showed a positive causal effect of opium use on time to death due to UGI cancer. Therefore, enhancing public knowledge and awareness about these destructive effects of opium can decrease the use of opium and reduce the amount of death due to UGI cancer.
